# Association between ventilatory settings and pneumothorax in extremely preterm neonates

**DOI:** 10.6061/clinics/2021/e2242

**Published:** 2021-03-15

**Authors:** Felipe Y. Matsushita, Vera L.J. Krebs, Werther B. de Carvalho

**Affiliations:** Departamento de Pediatria, Divisao de Neonatologia, Faculdade de Medicina FMUSP, Universidade de Sao Paulo, Sao Paulo, SP, BR.

**Keywords:** Air Leak Syndrome, Critical Care, Extremely Low Birth Weight Neonates, Mechanical Ventilation, Pneumothorax, Preterm

## Abstract

**OBJECTIVES:**

Pneumothorax is a catastrophic event associated with high morbidity and mortality, and it is relatively common in neonates. This study aimed to investigate the association between ventilatory parameters and the risk of developing pneumothorax in extremely low birth weight neonates.

**METHODS:**

This single-center retrospective cohort study analyzed 257 extremely low birth weight neonates admitted to a neonatal intensive care unit between January 2012 and December 2017. A comparison was carried out to evaluate the highest value of positive end-expiratory pressure (PEEP), peak inspiratory pressure (PIP), and driving pressure (DP) in the first 7 days of life between neonates who developed pneumothorax and those who did not. The primary outcome was pneumothorax with chest drainage necessity in the first 7 days of life. A matched control group was created in order to adjust for cofounders associated with pneumothorax (CRIB II score, birth weight, and gestational age).

**RESULTS:**

There was no statistically significant difference in PEEP, PIP, and DP values in the first 7 days of life between extremely low birth weight neonates who had pneumothorax with chest drainage necessity and those who did not have pneumothorax, even after adjusting for potential cofounders.

**CONCLUSIONS:**

Pressure-related ventilatory settings in mechanically ventilated extremely low birth weight neonates are not associated with a higher risk of pneumothorax in the first 7 days of life.

## INTRODUCTION

Pneumothorax is a catastrophic event with poor respiratory outcomes (1,2), high mortality (3,4), and is associated with a high rate of intraventricular hemorrhage (5,6). Pneumothorax occurs most commonly in newborns (7), especially in premature neonates. Among many risk factors, invasive mechanical ventilation is considered one of the most important risk factors for pneumothorax (2). There is great concern in using high pressures in invasive mechanically ventilated patients of this population, due to the risk of barotrauma because of their immaturity and low weight. High values of peak inspiratory pressure (PIP) and positive end-expiratory pressure (PEEP) are often associated with increased risk of pneumothorax (8). Due to its high morbidity and mortality, prevention of pneumothorax is crucial for better care for these newborns, and understanding which ventilatory parameters are associated with high risk of pneumothorax allows for a more careful ventilation, with adequate oxygenation, without exposing the newborn to the risk of pneumothorax. There have been no studies that evaluated ventilatory parameters and the risk of pneumothorax in extremely low birth weight neonates.

The aim of this study was to identify the association between pressure-related ventilatory settings (PEEP, PIP, and driving pressure [DP]) and pneumothorax with chest drainage necessity in extremely low birth weight newborns in the first 7 days of life. The hypothesis was that higher PEEP, PIP, and DP values may lead to an increased risk of pneumothorax in extremely premature newborns.

## MATERIAL AND METHODS

A single-center retrospective cohort study was conducted to analyze all newborns with birth weight less than 1,000 g, born between January 2012 and December 2017, and admitted to a tertiary neonatal intensive care unit in São Paulo, Brazil. Neonates who died in the delivery room and those with major malformations were excluded from the study. All data were collected by a review of each patient’s medical records, performed by a single researcher.

PEEP, PIP, and DP were documented every 3 hours in all neonates who received invasive mechanical ventilation. The driving pressure was calculated as (peak inspiratory pressure-positive end-expiratory pressure) and expressed in cmH2O. If the patient had 2 or more values of PEEP, PIP, or DP within the 3-hour interval, the highest value was considered. The patients were divided into two groups: group A (those without pneumothorax in the first 7 days of life), and group B (those with pneumothorax necessitating chest drainage in the first 7 days of life). All patients who had symptomatic pneumothorax underwent chest drainage according to the unit’s protocol. No patient underwent needle aspiration as the first-line approach.

For the group without pneumothorax, the highest values of PEEP, PIP, and DP within the first 7 days of life was considered. For the group with pneumothorax, the highest values of PEEP, PIP, and DP in the 24 hour-period before chest drain insertion was considered. As majority of the pneumothorax occurs in patients with birth weight less than 750 g, a subsequent analysis with only patients with birth weights less than 750 g was conducted. The primary outcome of the study was pneumothorax with chest drainage necessity in the first 7 days of life. A matched control group was created to adjust for cofounders associated with pneumothorax (CRIB II score, birth weight, and gestational age). We defined the “Match tolerance” as a CRIB II score of 3, birth weight of 100 g, and gestational age of 1 week.

Gestational age was determined using data from the last menstruation or obstetric ultrasound. Small for gestational age was defined as birth weight lower than the 10^th^ percentile, according to Fenton’s growth chart. The clinical risk index for babies (CRIB II) was used as a score to predict severity. All newborns were ventilated using Puritan Bennet^®^ 840 ventilators in pressure-controlled mode.

Results are presented as numbers with proportions or medians with interquartile ranges. All continuous variables were tested for normality using the Kolmogorov-Smirnov test. Comparisons between groups were performed using the Mann-Whitney U test for continuous variables and the chi-square test for categorical data. A receiver operating characteristic (ROC) curve was used to identify a threshold for ventilatory parameters and the risk of pneumothorax. After the ROC curve analysis, we used a cutoff for PEEP of 7 cmH2O, PIP of 20 cmH2O, and DP of 14 cmH2O, and divided them into two groups. All analyses were performed with IBM SPSS Statistics for Macintosh, Version 25.0. Statistical significance was set at *p*<0.05. The study protocol was approved and informed consent was obtained from the institution’s ethics committee (15762719.6.0000.0068 - CAPPESQ Comissão de Ética para Análise de Projetos de Pesquisa). The study was conducted in accordance with the strengthening the reporting of observational studies in epidemiology (STROBE) recommendations.

## RESULTS

During the study period, 279 newborns with birth weight less than 1,000 g who were admitted to the neonatal intensive care unit were identified. Twenty-two patients with major malformations were excluded from the study, resulting in a final population of 257 extremely premature neonates.

In the final population, the median gestational age was 27.1 (26-29.1) weeks and birth weight was 746 (600-880) g. The clinical characteristics of the newborns are shown in Table 1. We identified 26 (10.1%) as extremely low birth weight newborns who presented with pneumothorax with chest drainage necessity in the first 7 days of life. The group with pneumothorax had a lower gestational age, lower birth weight, and a higher CRIB II score, compared to the group that did not have pneumothorax in the first 7 days of life.

Regarding ventilatory parameters, none (neither PIP, PEEP, nor DP) was related to a higher rate of pneumothorax (Table 2). No ROC curve (for PEEP, PIP, or DP) showed an area under the curve with statistical significance (Table 3). Analyzing only neonates with birth weights less than 750 g, no ventilatory parameter related to pressure was significantly associated with a higher rate of pneumothorax (Table 4). A matched control group was created to adjust for cofounders associated with pneumothorax (CRIB II score, birth weight, and gestational age). The median CRIB II score of the matched-control group was 13 (12- 15), the median gestational age was 26.1 (25.1-27.3), and the median birth weight 665 (530-720), with no significant difference compared to the pneumothorax group. Likewise, there were no significant differences between the matched-control group and the pneumothorax group in terms of pressured-related ventilatory settings (Table 5).

## DISCUSSION

In this retrospective cohort study, PEEP, PIP, and DP were not related to an increased risk of pneumothorax in the first 7 days of life, even when adjusted for clinical characteristics associated with higher pneumothorax rates (CRIB II score, birth weight, and gestational age).

Pneumothorax is a relatively common condition in premature neonates (9), especially in the first days of life (10), and it is related to a high rate of mortality (4) and morbidities such as bronchopulmonary dysplasia and intraventricular hemorrhage. The first 7 days of life for a newborn of extremely low weight constitute a period of great fragility and instability (11). It is common for these newborns due to their immaturity to require invasive mechanical ventilation to maintain adequate oxygenation and ventilation, with relatively high mean airway pressure, especially if there was early fluid overload (12). However, due to their lung fragility, there is great concerns in using high pressures in invasive mechanical ventilation on these patients (13). Murphy et al. reported that mechanical ventilation is strongly associated with chest drainage insertion (14). Recently, a Cochrane review showed that in targeted-volume ventilation, there was a lower rate of pneumothorax compared with controlled pressure ventilation (15). Therefore, it can be inferred that tidal volume, together with lung compliance, and consequently the degree of lung distensibility, could be the major risk factors for air leak syndrome. That is, regardless of the PIP or PEEP values, as long as a high tidal volume and consequently alveolar hyperdistention is not produced, taking into account a homogeneous lung, there would be no increased risk of pneumothorax.

These data suggest that to avoid pneumothorax, strict tidal volume control should be performed rather than restricting pressure. In addition, new techniques for monitoring lung aeration are emerging, such as the use of point-of-care ultrasound (POCUS) (16). For this purpose, using a ventilatory mode such as volume-targeted ventilation could be beneficial. This ventilation mode has several other proven benefits, such as lower rate of mortality, bronchopulmonary dysplasia, severe intraventricular hemorrhage, and reduction in the duration required for mechanical ventilation (17).

To our knowledge, there are no studies to date assessing ventilatory settings and the risk of pneumothorax in extremely low birth weight newborns. Ventilatory settings data were accurately documented every 3 hours and no patients were excluded due to lack of data. However, this study has some limitations. First, this study is a retrospective and single-center study, a prospective and multi-center study is necessary to better assess the impact of ventilatory settings on this population. Secondly, the Puritan Bennett^®^ 840 Ventilator volume sensor is a distal one, meaning that the tidal volume measured is not reliable, especially in extremely premature neonates. A new study evaluating the tidal volume is necessary. Thirdly, due to the difficulty in evaluating the plateau pressure in this population, the DP was calculated utilizing PIP.

## CONCLUSION

This study suggests that pressure-related ventilatory settings (PEEP, PIP, and DP) are not associated with an increased risk of pneumothorax in the first 7 days of life in extremely low birth weight neonates.

## AUTHOR CONTRIBUTIONS

Matsushita FY conceptualized the study, drafted the initial manuscript, collected data, conducted the initial analysis, contributed to the interpretation of the results, and reviewed the manuscript. Krebs VLJ and Carvalho WB conceptualized the study, coordinated and supervised the data collection, contributed to the interpretation of the results, and reviewed the manuscript. All authors approved the final version of the manuscript as submitted and agree to be accountable for all aspects of the work.

## Figures and Tables

**Table 1:** Comparison of clinical characteristics between neonates with pneumothorax and those without pneumothorax in the first 7 days of life.

Parameter	Overall (n=258)	No pneumothorax (n=231)	Pneumothorax (n=26)	*p-*value
Gestational age (wk), median (IQR)	27.1 (26-29.1)	27.2 (26.1-29.2)	26.1 (24.2-27.1)	**0.001** a
Birth weight (g), median (IQR)	746 (600-880)	760 (605-892)	605 (520-700)	**0.001** a
CRIB II score, median (IQR)	12 (10-14)	11 (10-14)	14.5 (11-16)	**<0.001** a
Antenatal corticoid, n (%)	129 (62)	116 (50.2)	13 (50)	0.200^b^
Maternal chorioamnionitis, n (%)	29 (11.3)	27 (11.7)	2 (7.7)	0.541^b^
Small for gestational age, n (%)	125 (48.6)	113 (48.9)	12 (46.1)	0.789^b^
Female gender, n (%)	123 (47.9)	111 (48.1)	12 (46.1)	0.854^b^
APGAR 5 score, median (IQR)	8 (6-9)	8 (6-9)	7 (5-8)	0.073^a^
Vaginal birth, n (%)	41 (16)	34 (14.7)	7 (26.9)	0.107^b^
Twin birth, n (%)	77 (30)	66 (28.6)	11 (42.3)	0.147^b^

a Mann-Whitney U Test

b Chi-square.

**Table 2:** Comparison of ventilatory settings between the group with pneumothorax and the group without pneumothorax in the first 7 days of life.

Ventilatory setting	Overall	No pneumothorax	Pneumothorax	*p-*value
Highest peak inspiratory pressure (cmH2O), median (IQR)	19 (18-21)	18.5 (18-21)	19 (17-21)	0.991^a^
Peak inspiratory pressure>20 cmH2O, n (%)	62 (29.7)	53 (22.9)	9 (34.6)	0.120^b^
Highest PEEP (cmH2O), median (IQR)	7 (6-8)	7 (6-8)	7 (6-7)	0.984^a^
Highest PEEP>7 cmH2O, n (%)	54 (25.8)	48 (20.7)	6 (23)	0.120^b^
Highest driving pressure (cmH2O), median (IQR)	12 (11-14)	12 (11-14)	12 (10.5-13.5)	0.965^a^
Driving pressure>14 cmH2O, n (%)	46 (22)	41 (17.7)	5 (19.2)	0.118^b^

a Mann-Whitney U Test

b chi-square

**Table 3:** Receiver operating characteristic (ROC) area under the curve for highest PIP, PEEP and DP during the first 7 days of life.

Parameter	Area under curve (95% CI)	*p-*value
Highest peak inspiratory pressure	0.494	0.919
Highest PEEP	0.475	0.680
Highest driving pressure	0.439	0.320

CI: Confidence interval.

**Table 4:** Comparison of ventilatory settings between the group with pneumothorax and the group without pneumothorax in the first 7 days of life, among neonates with birth weight less than 750 grams.

Ventilatory setting	Overall (N=130)	No pneumothorax (N=108)	Pneumothorax (N=22)	*p-*value
Highest peak inspiratory pressure (cmH2O), median (IQR)	19 (18-21)	19 (18-21)	19 (17-22)	0.943^a^
Peak inspiratory pressure>20 cmH2O, n (%)	39 (30)	31 (28.7)	8 (36.3)	0.649^b^
Highest PEEP (cmH2O), median (IQR)	7 (6-8)	7 (6-8)	7 (6-7.5)	0.864^a^
PEEP>7 cmH2O, n (%)	35 (26.9)	29 (26.8)	6 (27.2)	0.766^b^
Highest driving pressure (cmH2O), median (IQR)	12,5 (11-14)	13 (12-14)	12 (11-13.5)	0.358^a^
Driving pressure>14 cmH2O, n (%)	27 (20.7)	23 (21.3)	4 (18.2)	0.696^b^

a Mann-Whitney U test

b chi-square test

**Table 5:** Matched control group comparison, adjusted for gestational age, birth weight and CRIB II score.

Parameter	No pneumothorax (n=26)	Pneumothorax (n=26)	*p-*value
Gestational age (wk), median (IQR)	26.1 (25.1-27.3)	26.1 (24.2-27.1)	0.777^a^
Birth weight (g), median (IQR)	665 (530-720)	605 (520-700)	0.667^a^
CRIB II score, median (IQR)	13 (12-15)	14.5 (11-16)	0.342^a^
Antenatal corticoid, n (%)	11 (50)	13 (50)	0.659^b^
Maternal chorioamnionitis, n (%)	6 (23.1)	2 (7.7)	0.124^b^
Small for gestational age, n (%)	11 (42.3)	12 (46.1)	0.780^b^
Female gender, n (%)	14 (53.8)	12 (46.1)	0.579^b^
APGAR 5 score, median (IQR)	8 (5-9)	7 (5-8)	0.354^a^
Vaginal birth, n (%)	8 (30.8)	7 (26.9)	0.760^b^
Twin birth, n (%)	7 (26.9)	11 (42.3)	0.244^b^
Highest peak inspiratory pressure (cmH2O), median (IQR)	19 (18-22)	19 (17-21)	0.639^a^
Peak inspiratory pressure>20 cmH2O, n (%)	9 (36)	9 (34.6)	1.000^b^
Highest PEEP (cmH2O), median (IQR)	7 (7-8)	7 (6-7)	0.082^a^
Highest PEEP>7 cmH2O, n (%)	10 (40)	6 (23)	0.479^b^
Highest driving pressure (cmH2O), median (IQR)	12 (11-15)	12 (10.5-13.5)	0.358^a^
Driving pressure>14 cmH2O, n (%)	7 (28)	5 (19.2)	0.803^b^

a Mann-Whitney U test

b chi-square test.
